# Clinical Relevance of ABCB1, ABCG2, and ABCC2 Gene Polymorphisms in Chronic Myeloid Leukemia Patients Treated With Nilotinib

**DOI:** 10.3389/fonc.2021.672287

**Published:** 2021-05-13

**Authors:** Federica Loscocco, Giuseppe Visani, Annamaria Ruzzo, Irene Bagaloni, Fabio Fuligni, Sara Galimberti, Antonello Di Paolo, Fabio Stagno, Patrizia Pregno, Mario Annunziata, Antonella Gozzini, Sara Barulli, Elisa Gabucci, Mauro Magnani, Alessandro Isidori

**Affiliations:** ^1^ Hematology and Hematopoietic Stem Cell Transplant Center, AORMN, Pesaro, Italy; ^2^ Department of Biomolecular Sciences, University of Urbino “Carlo Bo”, Fano, Italy; ^3^ Genetics and Genome Biology, Paediatric Laboratory Medicine (PLM), The Hospital for Sick Children, Toronto, ON, Canada; ^4^ Department of Clinical and Experimental Medicine, University of Pisa, Pisa, Italy; ^5^ AOU Policlinico Vittorio Emanuele, Divisioni Clinicizzata di Ematologia con Trapianto di Midollo Osseo, Catania, Italy; ^6^ AOU Città Della Scienza e Della Salute di Torino, Hematology, Torino, Italy; ^7^ Hematology, Cardarelli Hospital, Napoli, Italy; ^8^ AOU Careggi, Unità funzionale di Ematologia, Firenze, Italy

**Keywords:** chronic myeloid leukemia, nilotinib, drug resistance, MDR-ABC transporters, polymorphisms, molecular response

## Abstract

Tyrosine kinase inhibitors (TKIs) have radically changed the outcome of chronic myeloid leukemia (CML) patients in the last 20 years. Moreover, the advent of second generation TKIs, namely nilotinib and dasatinib, have largely increased the number of CML patients achieving deep and sustained molecular responses. However, the possible mechanisms capable of influencing the maintenance of the long-term molecular response are not yet fully known and understood. In this light, polymorphisms in MDR-ABC transporters may influence the efficacy and safety of TKIs. In this study, we examined seven single nucleotide polymorphisms (SNPs) in four ABC transporter genes: ABCC1 rs212090 (5463T>A), ABCC2 rs3740066 (3972C>T), ABCC2 rs4148386 G>A, ABCC2 rs1885301 (1549G>A), ABCG2 rs2231137 (34G>A), ABCG2 rs2231142 G>C, ABCB1 rs1045642 (3435C>T), to determine their effect on the achievement and/or loss of molecular response in 90 CML patients treated with nilotinib. We found that *ABCC2* rs3740066 CC and CT as well as the *ABCB1* rs1045642 TT genotypes correlated with a higher probability to achieve MR3 in a shorter time (*p*=0.02, *p*=0.004, and *p*=0.01), whereas *ABCG2* rs2231137 GG was associated with lower probability of MR3 achievement (*p*=0.005). Moreover, *ABCC2* rs3740066 CC genotype, the *ABCB1* rs1045642 CC and TT genotypes were positively correlated with MR4 achievement (*p*=0.02, *p*=0.007, and *p*=0.003). We then generated a predictive model incorporating the information of four genotypes, to evaluate the combined effect of the SNPs. The combination of SNPs present in the model affected the probability and the time to molecular response. This model had a high prognostic significance for both MR3 and MR4 (*p*=0.005 and *p*=0.008, respectively). Finally, we found *ABCG2* rs2231142 GG genotype to be associated with a decrease risk of MR3 loss. In conclusion, MDR-transporters SNPs may significantly affect the achievement and loss of molecular response in CML patients treated with nilotinib.

## Introduction

Targeted therapy with the selective inhibitors of BCR-ABL tyrosine kinase activity has dramatically changed the treatment of chronic myeloid leukemia (CML) in the last 20 years (1). As a result, patients treated with currently available tyrosine kinase inhibitors (TKIs) can now enjoy a near normal life-expectancy (2).

Imatinib mesylate (IM), a first generation TKI, has proven to be highly active and safe, even in the long run, in CML patients. Accordingly, it has become in recent years the golden standard for front-line CML treatment (3). Despite excellent results in terms of response, leukemic residual cells are still detectable in most patients with CML even after years of IM treatment. Some of these patients, especially those with advanced-phase CML, will eventually develop resistance to IM therapy, and need a second or a third line treatment (3, 4). The mechanisms of drug resistance have been extensively studied and can be classified as primary (also referred to as “refractoriness”), in which TKIs fail to induce an adequate response from the start of therapy, or acquired, developed after the initial achievement of some degree of response to IM, lasting for some time (4, 5). Second-generation BCR-ABL inhibitors (2GTKIs) have been initially developed to overcome acquired resistance to IM. Subsequently, 2GTKIs were tested frontline (2), becoming an alternative to frontline IM in intermediate and high-risk patients. In the path to cure for CML patients, a relevant problem is represented by the high discontinuation rate during treatment with 2GTKIs after IM failure. In the clinical setting, more than 50% of patients will eventually discontinue 2GTKIs treatment during their lifetime, due to lack/loss of response, toxicity or secondary resistance. Although point mutations in BCR-ABL kinase domain are the major mechanism of resistance to TKIs, the bio-availability of TKIs in leukemic stem cells is also an important pharmacokinetic factor that may contribute to its development (6).

The pharmacokinetic of TKIs is dependent on gastrointestinal absorption, metabolism and cellular influx and efflux (5). ATP-binding cassette (MDR-ABC) transporters are membrane glycoproteins that play a key role in the efflux of toxic agents, including TKIs, and are involved in the regulation of intracellular drug accumulation. Accordingly, changes in the level of expression and functionality of MDR-ABC transporters influence the efficacy and safety of the drug transported. ATP-binding cassette sub-family B member 1 (also known as P-glycoprotein MDR1) encoded by *ABCB1* gene, and ATP-binding cassette transporter G2 (also known as breast cancer resistance protein, BCRP) encoded by *ABCG2* gene, are two adenosine triphosphate-binding cassette members, with a prominent role in multidrug resistance (7). They are involved in the transport of bile, lipoprotein, drug substrates and other peptides. They are normally localized in the small intestine, in bile canalicular membranes of hepatocytes in the liver, in renal proximal tubes and in the blood-brain barrier. They are also abundantly expressed in hematopoietic progenitor cells, and overexpressed in cancer stem cells (8). As an efflux pump localized on the cellular plasma membrane, ABCB1, and ABCG2 proteins excrete a variety of endogenous and exogenous compounds including chemotherapeutic agents, such as mitoxantrone and methotrexate (9). Moreover, several recent findings support ABCB1 and ABCG2-mediated efflux of asciminib, a new drug in development in CML, as the mechanism of resistance in these cell lines (10, 11). In addition, multidrug resistance proteins 1 (MPR1) and 2 (MPR2), which are encoded by *ABCC1* and *ABCC2* genes, respectively are mostly involved in the transport of xenobiotics, and also play a role in excretion of TKIs (9). The overexpression of MDR-ABC transporters genes such as ABCB1, ABCG2, and ABCC2 has been reported to lead to interindividual difference in bioavailability of drugs, and it represents a potential mechanism of primary resistance to anti-cancer agents (5).

Genetic constitution of any individual plays a significant role in interpatient variability to therapeutic response. The genetic variations like single nucleotide polymorphisms (SNPs) can affect gene expression or function in normal and cancer cells causing inherent interindividual differences in the metabolism and in the availability of drugs (12). *ABCB1* gene, located at chromosome 7q21 is highly polymorphic; three polymorphisms have been extensively studied, two synonymous SNPs rs1128503 (1236C>T) in the exon 12 and rs1045642 (3435C>T) in exon 26; and one missense SNP rs2032582, (Ser893Ala/Thr 2677G>T/A) in exon 21. These three polymorphisms define a haplotype related to an increased activity in MDR1 (7). Moreover, several *ABCG2* DNA variants have been shown to reduce its protein expression through an effect of the drug that interacts with BCRP transporter (9, 12). A number of *ABCC2* SNPs have been studied for their potential functional influence on the expression level and transport activity. Few genetic variants −24C>T, 1249G>A and 3972C>T of *ABCC2* gene have been reported to lead to differences in pharmacokinetics of various drugs^7^. In the present study, we hypothesized that the most relevant SNPs in genes encoded for MDR1, BCRP, and MPR2 could predict the outcome of CML patients treated with nilotinib. The aim of this study was to evaluate whether the SNPs in *ABCB1, ABCC1, ABCC2*, and *ABCG2* genes have an impact on the efficacy of nilotinib treatment.

## Methods

### Study Population

90 Caucasian adult CML patients treated at six Italian sites were enrolled in an observational, multi-institutional, no-profit study (EudraCT: 2011-00637-37) from April 2011 to December 2017. All patients signed informed consent. The study was performed in accordance with the International Conference on Harmonization Good Clinical Practice Guidelines, the Declaration of Helsinki (1996) and it was approved by the Ethics Committees of all sites involved in the study.

To be eligible for inclusion, patients had to be at least 18 years of age, affected by CML in chronic phase requiring treatment with nilotinib either in first or second line. According to International scale^13^ major molecular response (MMR) was defined as BCR-ABL qPCR transcript level ≤0.1%, whereas MR4 indicated levels of disease that were ≤0.01% BCR-ABL transcript. Minimum follow-up to be included in the study was 12 months after the first dose of nilotinib.

Gene analyses were performed on DNA isolated from peripheral blood samples of patients screened in eight Italian Hematology Division. Blood samples were collected before nilotinib (baseline).

### Genotyping Analysis

This study examined seven SNPs in four ABC transporter genes: *ABCC1* rs212090 (5463T>A), *ABCC2* rs3740066 (3972C>T), *ABCC2* rs4148386 G>A, *ABCC2* rs1885301 (1549G>A), *ABCG2* rs2231137 (34G>A), *ABCG2* rs2231142 G>C, *ABCB1* rs1045642 (3435C>T). The genotyping was performed blind to the patients’ treatment and clinical outcomes. Genomic DNA was isolated from 1 ml of peripheral blood by means of commercially available kit (QIAamp DNA Blood Midi kit, Hilden, Germany) according to the manufacturer’s instructions. Polymorphisms for *ABCC1*, *ABCC2*, and *ABCG2* genes were detected by PCR high-resolution melting assay (HRM), restriction fragment length polymorphism (RFLP) of PCR products and pyrosequencing assays. The PCR for RFLP and pyrosequencing analyses were performed in a volume of 25 μl using 2 × PCR Master Mix kit (Diatheva, Fano, Italy), 25 ng of genomic DNA and 200 nM of each primer. The HRM assays were performed by using commercial kits according to the manufacturer’s instructions (Diatheva, Fano, Italy). Genotypes for ABCB1 rs1045642 (c.3435C>T) polymorphism were obtained through allele-specific probes using an Applied Biosystems kit on an ABI

Prism 7900HT Sequence Detection System instrument (ThermoFisher). Primer sequences, preparative PCR conditions and additional details are listed in **Table 1**.

**Table 1:** SNP, primer sequences and preparative PCR conditions.

Gene	SNP	Region	SNP ID	Primer sequence (5’ 3’)	PCR condition	Type of assay (RFLP by RE, HRM or pyrosequencing
ABCC1	5463T>A	3’-UTR	rs212090	F: ACTCCAGGCTTTCCCTTTTT R: TCCCATGAGGCCATCTCCTT	Preheat 95°C 10’ Den. 95°C 15’’ Ann. 58°C 30’ Ext. 72°C 30’’ Cycles: 35	RFLP RE: MboI T: 115 bp* A: 63 bp+52 bp*
ABCC2	3972C>T	exon28	rs3740066	F: TGAGCTGGATCTGGTCCTCA R: CTCCACCTACCTTCTCCATGC	Preheat 95°C 10’ Den. 95°C 5’’ Ann. 64°C 10’ Ext. 72°C 20’’ Cycles: 40 95°C 1’ 50°C 1’ HRM: Increase from 75°C to 85°C by steps of 0,2°C for 10” each	HRM
	T>C	intron	rs4148386	F: TCCCCAGCAGTTTCCAAGAC R: TTGGTAGTCCTGCATGCAGC	Preheat 95°C 10’ Den. 95°C 5’’ Ann. 64°C 10’ Ext. 72°C 20’’ Cycles: 40 95°C 1’ 50°C 1’ HRM: Increase from 75°C to 85°C by steps of 0,2°C for 10” each	HRM
	1549G>A	5’-Flank	rs1885301	F: [Btn]TAGTGTATGTTTGCTATTGAGTTGTA R: AAAAGGCAGCATTCAGTG Seq: ATGAAGAGTTAATATCCACA	Preheat 95°C 10’ Den. 95°C 15’’ Ann. 52°C 20’ Ext. 72° C 30’’ Cycles: 35	pyrosequencing
ABCG2	34G>A	exon2	rs2231137	F: TTGCAATCTCATTTATCTGGACTA R: [Btn]TTCAGTAAATGCCTTCAGGTCA Seq1: TCGAAGTTTTTATCCCA	Preheat 95°C 10’ Den. 95°C 15’’ Ann. 60°C 30”‘ Ext. 72°C 30’’ Cycles: 40	pyrosequencing
	C>A	exon5	rs2231142	F: [Btn]ACTGCAGGTTCATCATTAGCTAGA R: TTTTCCACATTACCTTGGAGTCTG Seq: AAGAGCTCCTGAGAACT	Preheat 95°C 10’ Den. 95°C 15’’ Ann. 64°C 30’ Ext. 72°C 30’’ Cycles: 40	pyrosequencing
ABCB1	3435C>T	exon 26	rs1045642	Probes: VIC TGTTGGCCTCCTTTGCTGCCCTCAC**[A]**ATCTCTTC-CTGTGACACCACCCGGC FAM TGTTGGCCTCCTTTGCTGCCCTCAC**[G]**ATCTCTTC-CTGTGACACCACCCGGC	Preheat 95°C 10’ Den. 95°C 10’’ Ann./Ext. 60°C 60” Cycles: 40	Real-time PCR**

Ann., annealing; Den., denaturation; Ext., extension; RE, restriction enzyme; SNP, single-nucleotide polymorphism; HRM, high-resolution melting; RFLP, restriction fragment length polymorphism.

*Length of amplicons after RE digestion.

**TaqMan SNP Genotyping Assay, cat no. C:_7586657_20 (ThermoFisher).

The gene expression levels of BCR-ABL1 were determined using quantitative real-time polymerase chain reaction (qRT-PCR) from total leukocyte RNA of peripheral blood samples. Ratios derived from BCR-ABL/ABL1 were converted to the International Scale (13).

### Statistical Analysis

All statistical analyses presented and discussed here were performed using Statistical Package for the Social Sciences SPSS version 20 (SPSS, Chicago, USA). The frequency of each genotype was calculated. Hardy-Weinberg equilibrium was verified for all examined SNPs. Age, sex, Sokal, Hasford and Eutos risk scores, were recorded. Demographic, as well as clinical data are presented as median and range for continuous variables or frequency (percentages) for categorical variables. Hematological, cytogenetic and molecular responses were assessed and classified according to ELN guidelines^13^. Furthermore, molecular monitoring was carried out as established by the European guidelines and applied by all Italian LABNET network (13, 14). Time to MR was calculated from nilotinib start to the achievement of a molecular response. The time to treatment failure was defined as the interval between the initiation of nilotinib therapy and the occurrence of event that indicated that patients had failed (loss of molecular response).

Demographic and clinical prognostic features were compared across response and SNPs, using Person’s χ^2^ test for categorical variables and Mann-Whitney test and Kruskal-Wallis tests for continuous variables, where appropriate.

The association between the cumulative probability to obtain or lost MR3 and MR4 among genotypes or haplotypes was calculated in univariate and multivariate analysis. Univariate analysis was performed with Kaplan-Meier method to establish the relationship between SNPs and molecular response. Then, multivariate analysis was performed by COX regression model, describing as the hazard ratio and 95% confidence interval. Statistical significance was set at P<0.05.

## Results

### Patients Characteristics

In our cohort, a large majority of patients (74%) received nilotinib at 600 mg/day and the remaining 26% at 800 mg/die. The characteristics of patients are listed in **Table 2**.

**Table 2:** Patients characteristic.

CLINICAL CHARACTERISTIC	N	(%)
***Median age at diagnosis, years*** (range)	48 (18-74)	
***Median Age at Nilotinib, years*** (range)	50 (18-74)	
***Sex***		
Male	52	58
Female	38	42
***Sokal score***		
Low	30	33
Intermediate	33	37
High	25	28
nv	2	2
***EURO/Hasford***		
Low	50	56
Intermediate	31	34
High	6	7
nv	3	3
***EUTOS***		
Low	83	92
High	1	1
nv	6	7
***Nilotinb treatment***		
I Line	46	51
II Line	44	49
***CCA in Ph+***		
Yes	6	7
No	79	88
na	5	5
***Transcript types***		
b2a2	43	48
b2a2+b1a1	1	1
b2a2+e1a2	1	1
b3a2	40	45
b3a2+b2a2	2	2
e1a2	1	1
nv	2	2

nv, not evaluable; CCA, clonal chromosome abnormalities; na, not available.

Overall, 83 patients (92%) achieved MR3 during treatment, after a median time of 6 months (range, 2–82). Fourteen patients (15%) lost MR3 during treatment, with a median time of 14 months (mean 21 months, range 4–78). Sixty-seven (74%) patients receiving nilotinib achieved a DMR (deep molecular response defined as MR≥4) after a median time of 22 months (range 2–76).

Molecular response rates were not significantly affected by age or sex. The median observation time was 78 months.

We did not find any relationship between age, sex, type of transcript and the achievement or loss of molecular response.

### Association of ABCC1, ABCC2, ABCG2, and ABCB1 SNPs and MR Achievement

The genotype and allele frequencies for all SNPs and their association with response to nilotinib are summarized in **Tables 3** and **4**.

**Table 3:** Cox regression model for SNPs and MR3.

Variable	SNP	Genotype	Coef	Hazard Risk	95% CI for Hazard risk lower	95% CI for Hazard risk upper	P- value
EUTOS 1			1.214	3.367	1.03	10.95	0.0436
ABCC2 rs3740066	3972C>T	CT	1.348	3.848	1.52	9.70	0.0043
ABCC2 rs3740066	3972C>T	CC	1.263	3.537	1.18	10.53	0.0234
ABCG2 rs2231137	34G>A	GG	-1.028	0.358	0.17	0.73	0.0052
ABCB1rs 1045642	3435C>T	TT	1.012	2.751	1.23	6.13	0.0132

p = 0.005.

**Table 4:** Cox regression model for SNPs and MR4.

Variable	SNP	Genotype	Coef	Hazard Risk	95% CI for Hazard risk lower	95% CI for Hazard risk upper	P- value
ABCC2 rs3740066	3972C>T	CC	1.365	3.917	1.20	12.74	0.0232
ABCB1rs 1045642	3435C>T	CC	1.446	4.247	1.46	12.30	0.0077
ABCB1rs 1045642	3435C>T	TT	1.288	3.625	1.54	8.48	0.003

p = 0.008.

Treatment outcomes were compared according to each candidate genotype for the seven statistical end points. To assess the association between polymorphisms and molecular response, we initially performed a survival analysis using each single variable (genotype or SNPs) and the time to reach MR3, and MR4 (data not shown). We further investigated the prognostic significance of SNPs and genotype using multiple Cox regression analysis, considering age, sex, and prognostic score as well as all molecular responses (MR3 and MR4).

**Figure 1:**
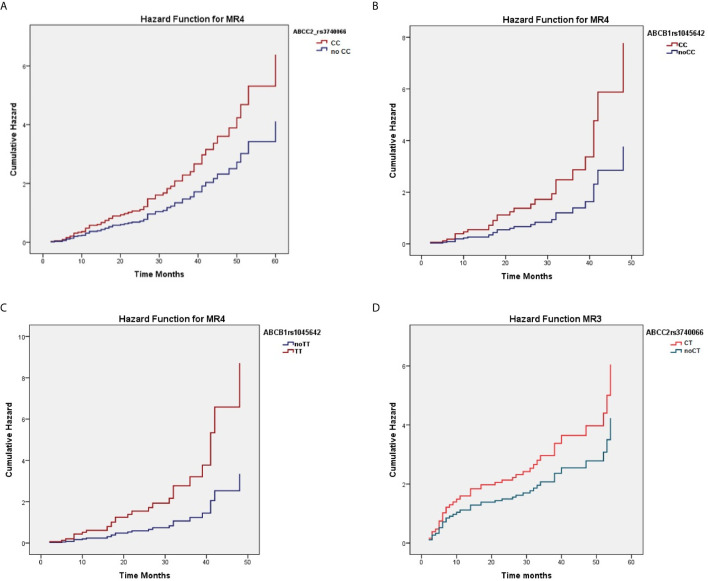
Correlation between *ABCC2* rs3740066 CC **(A)**, *ABCC2* rs3740066 CT **(B)**, *ABCB1* rs1045642 TT **(C)** *ABCG2* rs2231137 GG **(D)** genotypes and MR3 achievement.

**Figure 2:**
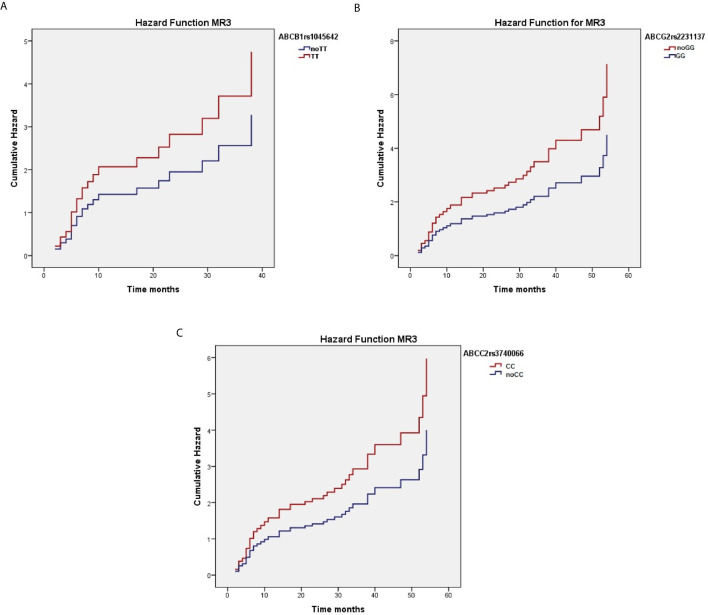
Correlation between ABCC2 rs3740066 CC **(A)**, ABCB1 rs1045642 CC **(B)** and TT **(C)** genotypes and MR4 achievement.

When considering the effect of the genotypes on treatment outcome, some SNPs were significantly associated with higher response rate to nilotinib therapy. As reported in **Table 3**, *ABCC2* rs374066, *ABCG2* rs2231137, and *ABCB1* rs1045642 SNPs affected the probability of achieving MR3. In details, the *ABCC2* rs3740066 CC and CT genotypes as well as the *ABCB1* rs1045642 TT genotype correlated with a high probability to achieve MR3 in a shorter time (*p*=0.02, *p*=0.004, and *p*=0.01) (**Figures 1A–C**). On the contrary, *ABCG2* rs2231137 GG genotype correlated with a lower probability of achieving a molecular response (*p*=0.005) (**Figure 1D**). The same analysis was done for MR4. *ABCC2* rs3740066 CC, *ABCB1* rs1045642 CC and TT genotypes showed a significant positive correlation with MR4 achievement (*p*=0.02, *p*=0.007, and *p*=0.003) (**Table 4**, **Figures 2A–C**).

We then performed a haplotype analysis for the studied genes, by analyzing the combined effect of several SNPs, and generated a predictive model incorporating four genotypes, risk score and MR3 (**Table 3**). The combination of SNPs (haplotypes) of the model affected the probability and the time to molecular response, with high prognostic significance (*p*=0.005). The same was done for MR4 (**Table 4**), and the high prognostic significance of the model was confirmed (*p*=0.008). We did not observe any significant association between *ABCC1* rs212090 (5463T>A) and MR achievement.

### Association of ABCC1, ABCC2, ABCG2, and ABCB1 SNPs and MR Loss

Subsequently, we analyzed the possible association between SNPs in *ABCC1*, *ABCC2*, *ABCG2*, and *ABCB1* genes and the loss of molecular response. We investigated whether the different distribution of genotypes or haplotypes were associated with MR loss during nilotinib treatment. *ABCG2* (rs2231142) GG genotype was associated with a decreased risk of MR3 loss, in a statistically significant fashion (**Table 5**).

**Table 5:** Cox regression model for SNPs and lost of MR3.

Variable	SNP	Genotype	Coef	Hazard Risk	95% CI for Hazard risk lower	95% CI for Hazard risk upper	P-value
ABCG2 rs2231142	G>T	GG	-1.296	0.274	0.09	0.80	0.018

p = 0.03.

## Discussion

CML patients treated with nilotinib show an improved clinical response, and most of them could reach treatment-free remission (TFR), once in deep and sustained molecular response. Several recent studies showed that TKIs can safely be discontinued, thus achieving a TFR (15–17). Moreover, TFR could be extended to patients who are in stable major molecular response (MMR), not necessarily MR4 (15). Nevertheless, a significant proportion of patients still fail to obtain or maintain a major MR (defined as ≥3 log reduction from standardized baseline) over time. For these patients, TFR will never become an achievable endpoint, and short and long term toxicities of nilotinib might thus represent a major flaw in everyday clinical practice. Although BCR/ABL mutations are the major contributory factor for the loss of response to TKIs (5), a reduced bio-availability of TKIs in leukemic stem cells may play a relevant role. In this regard, the presence of polymorphisms in drug transporters may contribute to mechanisms of drug resistance, disease progression or loss of MR. Therefore, the study of biomarkers predicting response to TKIs treatment may be of great help in treatment-decision making.

Pharmacokinetics of TKIs, has been proven to be a potential biomarker, and may partially explain the inter-individual variability in response to nilotinib (18, 19). The MDR-ABC family proteins are widely recognized for their ability to modulate the adsorption, distribution, metabolism, excretion of a broad range of compounds including TKIs. Polymorphisms in ABC transporters responsible for active drug efflux may have a role in predicting response to TKIs, even if the exact mechanism of MDR-ABC transporter-mediated resistance in CML is unclear (20).

In the current study, we examined whether genetic polymorphisms in *ABCB1*, *ABCC1*, *ABCC2*, and *ABCG2* genes may have an impact on the outcome of CML patients treated with nilotinib. ABC transporters, in particular ABCB1 and especially ABCG2, are abundantly expressed in hematopoietic progenitor cells and are overexpressed in cancer and CML stem cells (8). We observed that the SNPs of the major multi-drug resistance genes, *ABCB1*, *ABCC2*, and *ABCG2*, encoding for important export pumps expressed at tissue barriers, influenced the achievement of MR3 and MR4. In our study, patients with the variant alleles of either *ABCB1* rs1045642 or *ABCC2* rs3740066 achieved significantly higher rates of MR3 and MR4, in a shorter period of time. Conversely, *ABCG2* rs2231137 GG genotype predicted a lower MR3 rate, whereas *ABCG2* rs2231137 CC genotype did not. These findings suggest that SNPs in *ABCB1*, *ABCG2*, and *ABCC2* MDR genes seems to modulate the molecular response to nilotinib.

Many studies reported that the plasma levels of TKIs are associated with response to therapy in CML patients (21, 22). Clinical studies have established that ABCB1 expression levels are high in advanced stages of CML, and that high ABCB1 expression is associated with lower MMR rate and resistance to TKIs (23, 24).

Recently, Rinaldetti et al. analyzed the data of the Euro-SKY trial, in order to evaluate the expression of ABCG2, OCT1, and ABCB1, and their influence on TFR (25). Interestingly, after TKI discontinuation, ABCB1 expression was significantly downregulated in 40 patients, at the day of relapse. Furthermore, CML patients in deep molecular response showed a different expression in influx and efflux transporters after long-term imatinib treatment, compared with healthy controls. ABCB1 responded more sensitively to TKI exposure, because discontinuation of imatinib resulted in a significant downregulation or normalization of its transcription levels (25).

In another study evaluating the effect of ABCB1 variants on response to imatinib, Dulucq et al. showed that patients with rs1128503 TT or rs2032582TT/TA genotypes achieved significantly higher MMR rate (26). However, in a subsequent, larger cohort of patients, these results were not confirmed, questioning the real impact of ABCB1 variants on response to imatinib (27). Notwithstanding, Au et al. (19) described a significant association of ABCB1 rs1045642 CC genotype with both major and complete response in a Caucasian cohort of CML patients receiving imatinib. In an attempt to explain these discrepancies, it is important to underline that the site of polymorphism may be responsible for different conformation of the influx and efflux pumps, resulting or not in a clinically measurable effect on response to imatinib (19).

A different affinity toward ABCB1 has been reported for second generation TKIs,. Nilotinib has been implied to interact with ABCB1, but this interaction is controversial. Two studies demonstrated that nilotinib is a substrate of ABCB1 (28, 29); furthermore, 1) the increased expression of ABCB1 is associated with a reduced sensitivity of the K562 cell line to nilotinib; 2) ABCB1 and ABCC6 work in concert during the development of nilotinib resistance (30). Nilotinib is also a high-affinity substrate for ABCG2 and in higher concentrations, reduces both ABCB1 and ABCG2 ATPase activity (8). ABCG2 is expressed not only in HSCs but also in hepatocytes or intestinal epithelial cells, affecting the bioavailability of TKIs. Patients with a high ABGG2 expression were shown to have a two-fold increased risk of relapse (31). ABCB1 and ABCG2 were shown to have a significant impact on the cytotoxic potential of nilotinib, dasatinib and bosutinib *in vitro* (8). *In vitro*, K562 cells were efficiently protected from nilotinib cytotoxicity by ABCG2 activity, whereas dasatinib resistance was conferred by ABCB1 and ABCG2 activity. Notably, ABCB1 and ABCG2 only slightly modified the cytotoxic effect of bosutinib, suggesting a potential different mechanism of intracellular transportation for this latter. Finally, Desilly et al. (32) evaluated the impact of three common ABCB1 variants (1236C>T-2677G<T-3435>T) on the anti-proliferative effect and intracellular influx of nilotinib, dasatinib, imatinib and ponatinib in K562 and HEK 293 cell lines. Interestingly, ABCB1 polymorphisms were associated with response to imatinib, whereas they had a weak impact on the effects of nilotinib, dasatinib and ponatinib. However, these results were generated with cell lines, namely K562 and HEK293. Accordingly, major differences in outcome are expected, since the mechanisms concurring in drug-resistance in patients are different from cell lines.

Of note, our population did not comprise any patient with multi-TKI failure, a subpopulation of CML patients with the most challenging clinical risk factors in the TKI era (33). It would be interesting, in future studies, to see if there is any correlation between SNPS in ABCB1, ABCC1, ABCC2, and ABCG2, and multi-TKI resistant CML.

In conclusion, pharmacogenetic variability in individuals might predict treatment outcome in CML patients receiving nilotinib. Our results showed the effect of the SNPs studied on the molecular response to nilotinib, significantly predicting response to therapy for MR3 and MR4 achievement and loss. These models were highly predictive. The combined effect of ABCB1 and ABCG2 polymorphisms could be synergistic and promote the achievement and/or loss of the molecular response. Further studies in larger cohorts of patients are warranted to confirm our preliminary observation.

## Data Availability Statement

The original contributions presented in the study are included in the article/supplementary material. Further inquiries can be directed to the corresponding author.

## Ethics Statement

The studies involving human participants were reviewed and approved by CERM, Comitato Etico della Regione Marche. The patients/participants provided their written informed consent to participate in this study.

## Author Contributions

FL, GV, and AI designed the study and wrote the manuscript. AR, IB, SG, AP, and MM were responsible for biological studies. FS, PP, MA, AG, SB, and EG treated the patients, collected the data, and commented on the manuscript. FF performed statistical analysis. All authors contributed to the article and approved the submitted version.

## Funding

The study was supported in part by AIL Pesaro Onlus.

## Conflict of Interest

The authors declare that the research was conducted in the absence of any commercial or financial relationships that could be construed as a potential conflict of interest.
